# EFEMP1 is repressed by estrogen and inhibits the epithelial-mesenchymal transition *via* Wnt/β-catenin signaling in endometrial carcinoma

**DOI:** 10.18632/oncotarget.8263

**Published:** 2016-03-22

**Authors:** Tingting Yang, Huilin Zhang, Haifeng Qiu, Bilan Li, Jingyun Wang, Guiqiang Du, Chune Ren, Xiaoping Wan

**Affiliations:** ^1^ Department of Obstetrics and Gynecology, Shanghai First People's Hospital Affiliated to Nanjing Medical University, Nanjing, China; ^2^ Department of Obstetrics and Gynecology, Shanghai First Maternity and Infant Hospital, Tongji University School of Medicine, Shanghai, China; ^3^ Center for Reproductive Medicine, Affiliated Hospital of Weifang Medical University, Weifang, China; ^4^ Department of Gynecology and Obstetrics, Nanjing Maternal and Children Care Hospital Affiliated to Nanjing Medical University, Nanjing, China

**Keywords:** EFEMP1, endometrial carcinoma (EC), estrogen receptor α (ERα), epithelial-mesenchymal transition (EMT), Wnt/β-catenin

## Abstract

Epidermal growth factor-containing fibulin-like extracellular matrix protein 1 (EFEMP1) acted as a tumor suppressor in endometrial carcinoma (EC). However, the correlation between EFEMP1 and estrogen is unknown. Here, we reported that the expression of EFEMP1 was conversely associated with ERα in endometrial carcinoma tissues. In endometrial carcinoma cells, estrogen/ERα signaling significantly suppressed the expression of EFEMP1. Moreover, chromatin immunoprecipitation (CHIP) and dual-luciferase reporter assays demonstrate that estrogen/ERα bound to the estrogen response element (ERE) located in EFEMP1 promoter and repressed its expression. Besides, *in vitro* and *in vivo*, EFEMP1 could remarkably suppress the expression of epithelial-mesenchymal transition (EMT) markers such as Vimentin, Snail and the Wnt/β-catenin target genes like Cyclin-D1 and c-Myc, which could be restored when EFEMP1 was silenced. In addition, XAV93920 (the inhibitor of the Wnt/β-catenin pathway) blocked and LiCl (the activator of the Wnt/β-catenin pathway) enhanced the effect of EFEMP1 on EMT. In conclusion, we demonstrated that estrogen/ERα signal suppresses EFEMP1. Besides, EFEMP1 inhibits EMT via interfering the Wnt/β-catenin signaling.

## INTRODUCTION

Endometrial carcinoma (EC) is one of the most common cancers of the female reproductive system, with an estimated 54,870 new diagnosed cases and 10,170 deaths in the United States in 2015 [1]. The unopposed action of estrogen (E2) is the main etiological factor contributing to the tumorigenesis and progression of EC, and several studies have shown that EC tissue synthesized estrogen independent of the concentration in the serum to maintain high estrogen levels [2, 3]. Thus, it is necessary to further explore the mechanisms underlying the function of estrogen in EC [4].

Epidermal growth factor-containing fibulin-like extracellular matrix protein 1 (EFEMP1, or Fibulin-3) belongs to the fibulin family of extracellular glycoproteins characterized by a fibulin-type C-terminal domain and tandem calcium binding epidermal growth factor (EGF)-like modules [5]. EFEMP1 was initially described in senescent and Werner syndrome fibroblasts and has been reported to be associated with inherited forms of macular degeneration [6]. However, the role of EFEMP1 in tumorigenesis and cancer progression remains controversial. In pancreatic adenocarcinoma [7], EFEMP1 expression promoted tumor growth and metastasis. In cervical cancer, upregulation of EFEMP1 not only promoted angiogenesis but also associated with lymph node metastasis [8]. Inversely, in sporadic breast cancer and non-small cell lung cancer, EFEMP1 was reported to be silenced through hypermethylation of the promoter region and suppress cancer invasion [9, 10]. Recently, we reported that EFEMP1 was downregulated in EC in part due to aberrant promoter methylation [11], and we also noted that EFEMP1 was downregulated in EC without hypermethylation. A previous study revealed the regulatory elements in the promoter region of EFEMP1, including an estrogen response element (ERE), three Sp1 binding sites and a Tant motif [12]. Therefore, we hypothesized that estrogen may also regulate the expression of EFEMP1 in EC.

The epithelial-to-mesenchymal transition (EMT) is a developmental process characterized by loss of the epithelial homotypic adhesion molecule E-cadherin and the gain of mesenchymal markers, such as Vimentin and/or Fibronectin, which promotes tumor invasion and metastasis [13, 14]. Several signals from the tumor microenvironment have been shown to trigger EMT processes via specific pathways involving epidermal growth factor (EGF), transforming growth factor (TGF)-β, hepatocyte growth factor (HGF), Notch and Wnt/β-catenin signaling [15]. Previous research has implicated dysregulation of the Wnt/β-catenin pathway in many malignant tumors and highlighted the role of this pathway as an important regulator of EMT in cancer cells [16, 17]. Genetic or epigenetic alterations in the regulation of Wnt/β-catenin components led to aberrant activation of target genes, including c-Myc and Cyclin-D1, and promoted cell growth, invasion and metastasis [18]. In our previously study, we found that EFEMP1 acted as a tumor suppressor to inhibit cancer proliferation, invasion and migration. Moreover, aberrant expression of EFEMP1 was associated with downregulation of E-cadherin and increased expression of Vimentin [11]. These results suggest that EFEMP1 may suppress tumor invasion through inhibiting EMT in EC.

Here, we demonstrate that E2 suppresses EFEMP1 expression by directly binding to its promoter in EC. In addition, overexpression of EFEMP1 suppresses EMT and inhibits cell invasion and migration via Wnt/β-catenin pathway. This role of EFEMP1 in the etiology and progression of EC may provide a new drug target for future treatment.

## RESULTS

### EFEMP1 expression is downregulated by E2 in EC

**Figure 1:**
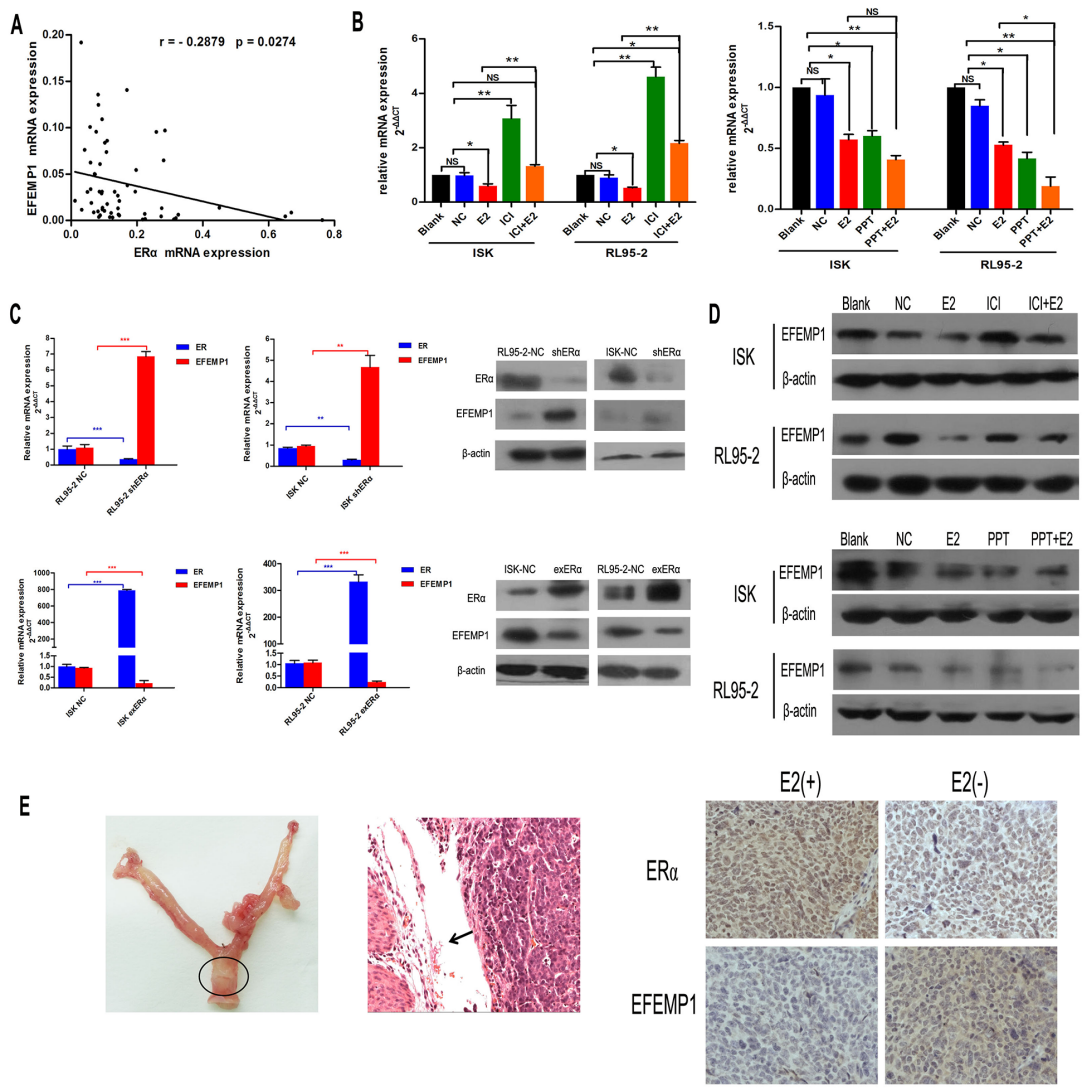
17β-estrogen inhibits the expression of EFEMP1 in EC tissues and cells **A.** Quantitative real-time PCR analysis of the association between EFEMP1 and ERα in 58 fresh samples of EC. **B.** Treatment of Ishikawa and RL95-2 cell lines with 1μM and 100nM 17β-estrogen for 9 h. The cells were treated with 17β-estrogen alone or were co-treated with 1μM ICI182780, an estrogen receptor agonist, or 1μM PPT, an estrogen receptor agonist for 7h. Then, EFEMP1 mRNA expression was analyzed using qRT-PCR. **C.** Ishikawa and RL95-2 cells were transfected with plasmids expressing shERα or exERα, respectively, for 48 h, the mRNA and protein levels of EFEMP1 and ERα were then assayed using qRT-PCR and Western blot. β-actin was used as an internal control (Ctrl). Error bars denote the mean ± SD of three independent experiments., *p<0.05, **p<0.01, ***p<0.001 compared to the ‘blank’ group and/or ‘NC’ group. **D.** The protein levels of EFEMP1 in Ishikawa and RL95-2 cells following treatment with 17β-estrogen, ICI182780 and/or PPT for 48 h were assayed using Western blotting. **E.** Characterization of an orthotopic endometrial cancer model (left). Histological examination showed that the tumor developed from the uterine cavity (arrow indicated for orthotopic tumor, middle). Effect of estrogen on EFEMP1 expression is analysed by immunohistochemical studies of ERα and EFEMP1(right). Original magnification ×400.

In our previous study, we observed that EFEMP1 was downregulated in some EC tissues without epigenetic changes and there was an ERE element at the promoter of EFEMP1. Therefore, our current study sought to further investigate the potential association between EFEMP1 and ERα. Firstly, we examined the expression of EFEMP1 and ERα in 58 EC samples (Figure 1A), and we found that EFEMP1 was inversely correlated with ERα expression at the mRNA level (r=−0.2879, P=0.0274). To further investigate whether EFEMP1 was regulated by E2, RL95-2 and Ishikawa cells were cultured in estrogen-depleted medium for 24 hours prior to drug treatment. As our data shown, the expression of EFEMP1 decreased after treatment with E2 and/or PPT (an ERα-selective agonist), and this effect was reversed after treatment with 1 μM ICI182780 (an ERα-selective antagonist, Figure 1B and 1D). To determine the role of ERα in the expression of EFEMP1, RL95-2 and Ishikawa cells were transfected with an ERα shRNA (sh-ERα) and ERα-expressing vector (ex-ERα) for 48 h. The results showed that silencing ERα increased the expression of EFEMP1 and overexpression of ERα decreased the expression of EFEMP1 (Figure 1C). In orthotopic tumor sections, we also found that E2 treatment group showed high level ERα and low expression of EFEMP1 (Figure 1E). Furthermore, our immunofluorescence data revealed the similar results (Supplementary Figure S1).

### EFEMP1 is a direct target of ERα

**Figure 2:**
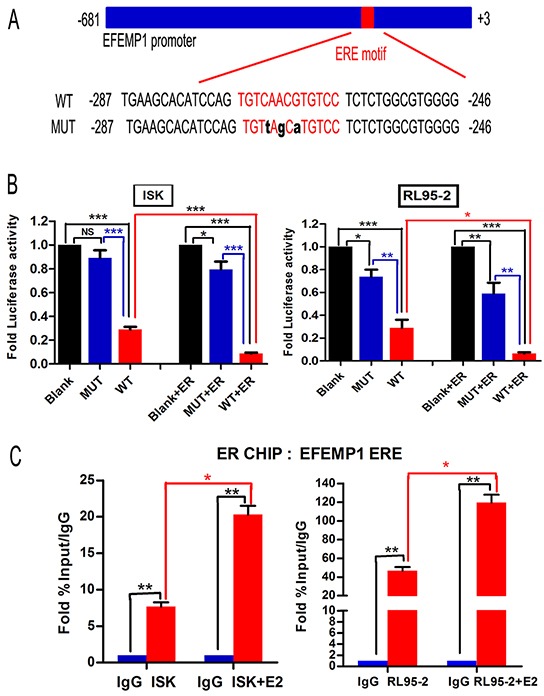
ERα binds to the EFEMP1 promoter and suppresses its expression in EC cells **A.** A schematic of the EFEMP1 promoter, showing the mutant binding site in black. Both of the promoter regions were cloned into the pGL3 reporter plasmid. **B.** Ishikawa and RL95-2 cells were transfected with 200 ng of the pGL3 promoter luciferase construct containing the EFEMP1 promoter regions as shown in (A), together with 200 ng of internal control plasmid expressing Renilla-luciferase. To further evaluate the role of ERα in EFEMP1 expression, cells were also co-transfected with ERα-expressing plasmids. Relative luciferase activities were measured at 48 h after transfection. The results are shown as the fold induction after normalizing to Renilla-luciferase activity. All experiments were performed at least twice, in triplicate. **C.** ChIP analysis of the physical association between ERα and the EFEMP1 promoter region. Crosslinked protein-chromatin complexes were immunoprecipitated from Ishikawa and RL95-2 cells using an anti-ERα antibody or a non-specific IgG control. Furthermore, cells were pretreated with E2 at 1μM or 100nM for 3 h to investigate the interaction between ERα and EFEMP1. The immunoprecipitated DNA was quantitated using real-time PCR. The enrichment of targeted genomic regions was assessed relative to the input DNA.

To elucidate the mechanism how does E2 mediate EFEMP1 expression, we performed ChIP assays using Ishikawa and RL95-2 cells, followed by RT-PCR using primer sets flanking the putative ERα binding sites in the core promoter region (Figure 2A). As shown in Figure 2B, ERα was significantly enriched in RL95-2 and Ishikawa cells by approximately 43-fold and 7-fold, respectively. After treating the cells with E2 for 3h, we noted 120-fold and 21-fold enrichment over the isotype antibody in RL95-2 and Ishikawa cells, respectively. These results support that ERα can directly bind to the ERE located in EFEMP1 promoter.

To further address whether ERα can repress the EFEMP1 promoter, we preformed dual-luciferase reporter assays in both RL95-2 and Ishikawa cells. We transfected WT or MUT (mutation of the ERE motif) EFEMP1 promoter reporter constructs into cells with or without co-transfection of an ERα expression vector. In WT group, both Ishikawa and RL95-2 cells showed significant reduction of approximately 71% and 75% in luciferase activity, respectively. After co-transfection with the ERα expression vector, WT groups of Ishikawa and RL95-2 showed the greatest reduction in activity, at approximately 91% and 94%, respectively. Whereas, mutations in the ERE sequence of EFEMP1 abolished the suppressive effects of ERα (Figure 2C). Collectively, these data suggest that ERα directly suppresses EFEMP1 expression in EC cell lines.

### Negative correlation of EFEMP1 and EMT-related proteins in clinical samples

**Figure 3:**
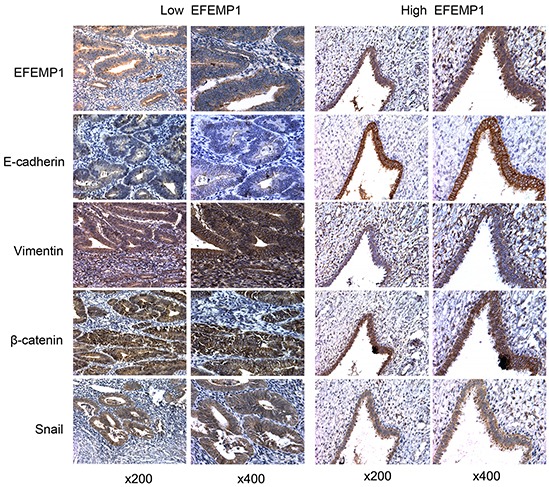
EFEMP1 expression correlates with EMT markers IHC staining of EFEMP1, E-cadherin, Vimentin, Snail and β-catenin in samples from patients with low and high levels of EFEMP1 expression. Magnification, x200 (left), x400 (right).

In our previous study, we noted that EFEMP1 was downregulated in EC and was associated with lymph node metastasis. In the current study, using IHC analyses, we observed that the expression of EFEMP1 and E-cadherin were significantly decreased in 120 EC samples (Supplementary Table S1) compared to 50 normal endometrium (P<0.001). In contrast, the expression of mesenchymal markers like Vimentin, Snail and β-catenin were much higher in EC samples (Table 1). Moreover, the expression of EFEMP1 positively correlated with E-cadherin (r=0.499, P<0.001) but inversely correlated with Vimentin, Snail and β-catenin (r=−0.164, −0.369 and −0.299, respectively, Table 2). Representative stainings for EMT-related proteins associated with varying expression levels of EFEMP1 were shown in Figure 3.

**Table 1:** Expression of EFEMP1, E-cadherin, Vimentin, Snail and β-catenin in 120 cases of endometrial carcinoma and 50 normal endometrium

	Endometrial carcinoma	Normal endometrium	P* value
EFEMP1			<0.001
Positive	32	45	
Negative	88	5	
E-cadherin			<0.001
Positive	52	42	
Negative	68	8	
Vimentin			0.019
Positive	65	17	
Negative	55	33	
Snail			<0.001
Positive	70	12	
Negative	50	38	
β-catenin			<0.001
Positive	67	7	
Negative	53	43	

* χ^2^ test.

**Table 2:** The correlation between the expression of EFEMP1 and E-cadherin, Vimentin, Snail and β-catenin in endometrial carcinoma tissues

Proteins	EFEMP1			
	Positive	negative	r	P*-value
E-cadherin			0.499	<0.001
positive	27	25		
negetive	5	63		
Vimentin			−0.164	0.097
positive	13	52		
negetive	19	36		
Snail			−0.369	<0.001
positive	9	61		
negetive	23	27		
β-catenin			−0.299	0.002
positive	10	57		
negetive	22	31		

* Spearman test.

### EFEMP1 suppresses EMT *in vitro* and *in vivo*

**Figure 4:**
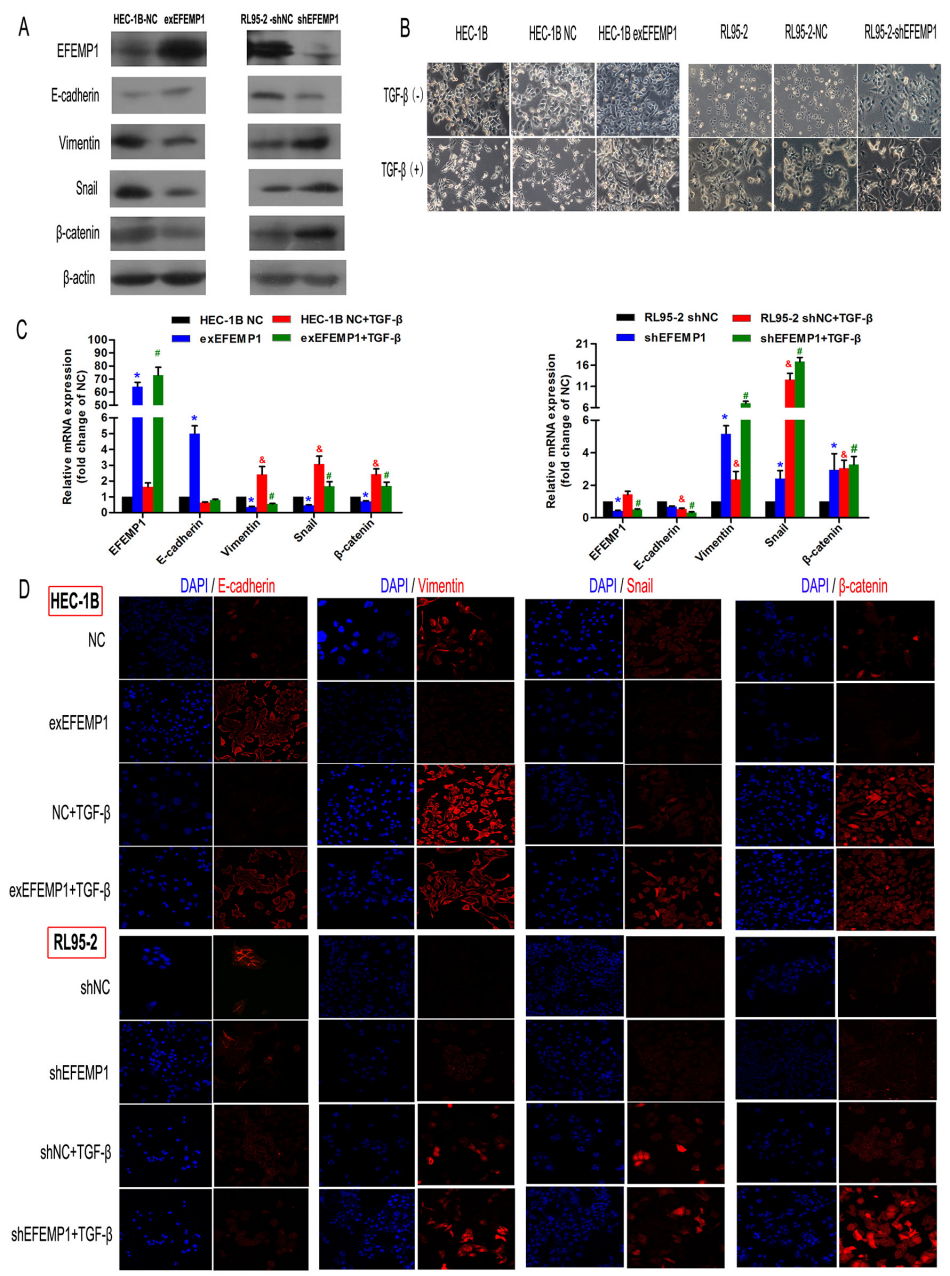
EFEMP1 suppresses EMT in EC cells **A.** Immunoblotting analysis of the expression of the four EMT-related markers (E-cadherin, Vimentin, Snail and β-catenin) in HEC-1B and RL95-2 cells transfected with plasmids expressing exEFEMP1 and shEFEMP1, respectively. β-actin was used as an internal control. **B.** The changes in cellular morphology induced by TGF-β that resulted in EMT were evaluated in different groups using phase contrast microscopy. **C.** Four cell types, HEC-1B-NC, HEC-1B-exEFEMP1, RL95-2-NC and RL95-2-shEFEMP1, were incubated for 48 h in the absence or presence of TGF-β (2 ng/ml) to induce EMT. Then, total RNA was isolated and subjected to quantitative real-time PCR to monitor the expression of EMT-related marker transcripts, which were normalized to β-actin levels. Data shown are the mean (±SD; n=3), *p<0.05, ^#^p<0.05, ^&^p<0.05 different group compared with the ‘NC group’. **D.** Immunofluorescence microscopic analysis of changes in the expression and localization of EMT markers. Cells (c) were untreated or treated with TGF-β for 3 days and stained with anti-E-cadherin, anti-Vimentin, anti-Snail or anti-β-catenin antibodies. Altered cytoskeletal architectures were identified using rhodamine-phalloidin immunofluorescence as indicated. Statistical values were calculated using t-tests, and experiments were performed at least three times.

To further investigated the role of EFEMP1 in EMT, we established stably transfected HEC-1B-exEFEMP1 and RL95-2-shEFEMP1 cells. Data showed that compared to the control group, knockdown EFEMP1 induced EMT-like morphological features, such as a spindle-shaped appearance (Figure 4B), and led to significant reduction in E-cadherin expression levels as well as increase in Vimentin, Snail and β-catenin expression levels (Figures 4A and 4C). However, enforced stable expression of EFEMP1 in HEC-1B (do not express detectable endogenous EFEMP1 [11]) led to an increased cuboidal appearance (Figure 4B). In agreement with the changes in cellular appearance, HEC-1B-exEFEMP1 stimulated the expression of E-cadherin and inhibited the expression of mesenchymal cell markers (Figures 4A and 4C).

Next, to directly test the function of EFEMP1 in EMT, we evaluated its effect on TGF-β-induced EMT. We observed that control cells underwent marked EMT modification within 72 h with TGF-β treatment, and EFEMP1-overexpressing cells kept their epithelial features (Figure 4B). Accordingly, E-cadherin expression was retained in EFEMP1-overexpressing cells but lost in control cells after treatment with TGF-β (Figures 4C and 4D). We also found that enforced EFEMP1 expression blocked the TGF-β-induced numerous mesenchymal genes, such as Vimentin, Snail and β-catenin. While in EFEMP1-silenced cells, TGF-β treatment induced more mesenchymal characteristics than control cells (Figures 4C and 4D).

**Figure 5:**
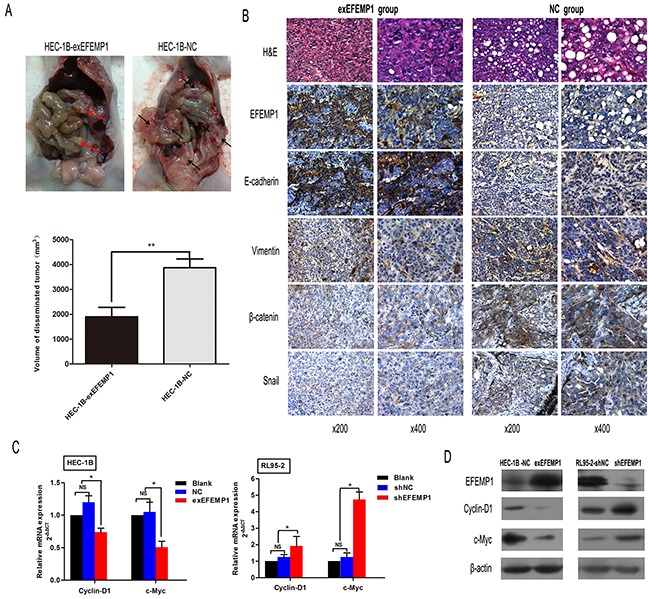
EFEMP1 downregulates the expression of target genes in the Wnt/β-catenin pathway and suppresses EMT in mice peritoneum metastasis model **A.** Effect of EFEMP1 on peritoneum metastasis tumor volume is expressed as mean ± SD of each group. **p<0.01. **B.** Representative examples of IHC staining of EMT markers in disseminated tumors from each group of mice. Magnification, x200 (left), x400 (right). **C.** The effect of EFEMP1 overexpression or inhibition on mRNA levels of the Wnt/β-catenin target genes Cyclin-D1 and c-Myc was analyzed using real-time PCR in the indicated EC cells. Error bars denote the SD calculated from three independent experiments. *p<0.05. **D.** Western blotting was performed to examine protein expression of the Wnt/β-catenin pathway target genes Cyclin-D1 and c-Myc in the indicated cells. β-actin was used as a loading control.

In nude mouse peritoneum metastasis model we further tested the suppressive role of EFEMP1 on EMT. We observed that the number and volume of disseminated tumors in the HEC-1B-exEFEMP1 group were significantly lower than the HEC-1B-NC group (P<0.001 and P<0.01, Figure 5A). Besides, we performed immunohistochemistry to test the expression of EFEMP1, E-cadherin, Vimenten, Snail and β-catenin on tumor sections. We found that Vimenten, Snail and β-catenin were decreased in HEC-1B-exEFEMP1group tumors, while E-cadherin was increased (Figure 5B). These results suggest that EFEMP1suppresses EMT in EC.

### EFEMP1 blocks Wnt/β-catenin signaling in EC

Due to its critical role in tumorigenesis and metastasis in EC, we next examined whether EFEMP1 could affect the Wnt/β-catenin signaling. In HEC-1B cells, EFEMP1 overexpression significantly suppressed the expression of Cyclin-D1 and c-Myc, which were target genes of Wnt/β-catenin signaling. Conversely, knockdown of EFEMP1 in RL95-2 cells could activated the expression of Cyclin-D1 and c-Myc (Figures 5C and 5D). These data suggest that EFEMP1 could block the Wnt/β-catenin pathway in EC.

### EFEMP1 suppresses EMT process through Wnt/-catenin signaling

**Figure 6:**
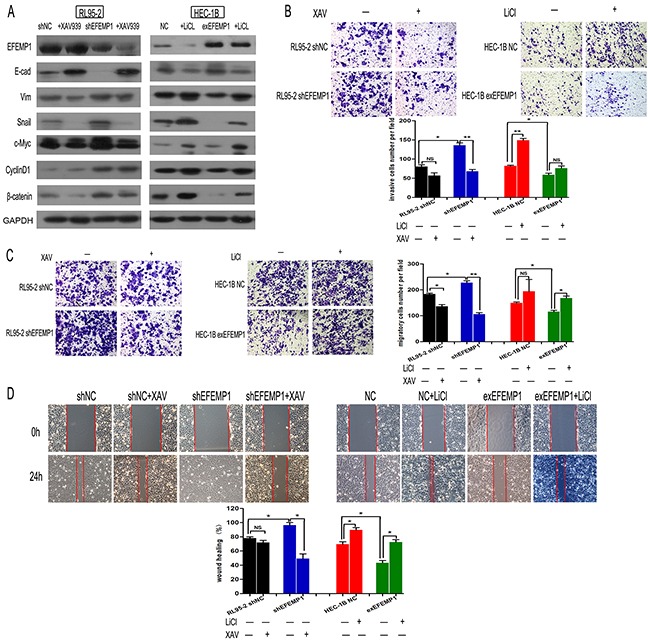
EFEMP1 suppresses EMT via the Wnt/β-catenin pathway **A.** Expression levels of EFEMP1, EMT-related markers and Wnt/β-catenin pathway downstream targets in the indicated cells following treatment with 10 μmol/l XAV93920 or 20 mmol/l LiCl for 24 h were examined using Western blotting. Glyceraldehyde 3-phosphate dehydrogenase was used as an internal control. **B.** The invasive capacity of different groups was measured using a transwell assay. Representative images were captured after LiCl or XAV93920 treatments for 48 h, and statistical significance was determined using the arithmetic means ± SD of three independent experiments. Magnification, x100. *p<0.05, **p<0.01. **C.** Migratory capacity was examined using a transwell assay without BD Matrigel. **D.** Graphical presentation of the percentage of wound-healing, which was calculated as (0 h width - 24 h width of wound) / (0 h width of wound); original magnification, x100. The percentage of wound closure was measured from at least three randomly selected regions (mean ± SD).

Then, to analyze the role of Wnt/β-catenin signaling during EFEMP1-inhibiting EMT, we used the Wnt/β-catenin pathway inhibitor XAV939 and activator LiCl. As expected, in RL95-2-shEFEMP1 cells, XAV939 markedly reduced the expression of Cyclin-D1 and c-Myc. While in HEC-1B-exEFEMP1 cells, LiCl could increase the expression of these genes (Figure 6A). In addition, activation of Wnt/β-catenin signaling by LiCl impaired the effect of EFEMP1-overexpressing on the suppression of EMT related markers. In contrast, inhibition of Wnt/β-catenin reversed the effect of knockdown EFEMP1 on EMT related proteins (Figure 6A). Furthermore, we examined the biological mechanism of EFEMP1 in EC. Through Transwell and Wound healing assay, we found that inhibition of Wnt/β-catenin signaling in RL95-2-shEFEMP1 could abrogate EFEMP1 silencing-induced invasion and migration. Moreover, activation of this pathway by treatment with LiCl promoted cell migration and invasion in HEC-1B-exEFEMP1 cells (Figure 6B, 6C and 6D). Taken together, our results indicate that Wnt/β-catenin signaling is a functional mediator of EFEMP1-related EMT and invasion in EC cell lines.

## DISCUSSION

Previous studies have shown that EFEMP1 regulates cancer in a context-dependent manner [19], and we have proven EFEMP1 acted as a tumor suppressor in EC. In the current study, we focused on the underlying mechanism of EFEMP1 in EC. In EC cells, we found that E2 downregulated EFEMP1 via binding to the ERE within its promoter. Furthermore, our results showed that EFEMP1 suppressed EMT through blocking the Wnt/β-catenin signaling, which further inhibited tumor invasion and migration in EC.

Recent study has shown that the concentration of E2 in endometrial carcinoma tissue was significantly higher than normal endometrium. Recently, *in situ* estrogen metabolism, including synthesis and degradation has been thought to play a very important role in the development and progression of EC. Qi Che *et al.* reported that there was activation of a positive feedback loop in EC, IL-6 stimulated by E2 in endometrial cancer cells induced aromatase expression in stromal cells, promoting enhanced intratumoral E2 synthesis [2, 20]. Data from our study showed an inverse relationship between EFEMP1 and ERα. The similar phenomenon had been reported in retinal diseases (drusen) [12] and breast cancer [21, 22]. However, there were no reports about the direct interactions of E2 and EFEMP1. Using CHIP and luciferase reporter assays, we demonstrated that estrogen/ERα signal could inhibit the expression of EFEMP1 by directly binding to the ERE motif localized in EFEMP1 promoter. Many of these E2-repressed genes are cell cycle inhibitors, proapoptotic genes or tumor suppressors; thus, their repression may be a critical step for cancer development and progression [23]. Endometrium has both α and β forms of the ER. However, the AF-1 (activating function-1) domain in ERβ was less transcriptional activation compared with the AF-1 domain of ERα. These differences in functional homology between ERα and ERβ might partially account for the differences in E2 responsiveness [24]. Although ERα was primarily considered a transcriptional activator, significant researches have demonstrated that more than half of all E2-regulated genes are instead repressed [25]. In view of the repressive role of ERα, mechanisms explaining this phenomenon included the removal of co-activators from promoters or enhancers [25, 26], co-repressor competition with co-activators at the regulatory regions of repressed genes [27] and active recruitment of repressive complexes, such as NCoR, SMRT or HDACs [28]. Despite these findings, the mechanisms underlying the repression of EFEMP1 by estrogen still requires further investigation in EC.

EMT is a multistage process that is required during embryonic development. However, increasing evidence suggests that this normal genetic program is often hijacked in various pathological conditions, such as tissue fibrosis and cancer metastasis [13, 29]. In our previous study, we showed that EFMEP1 expression in EC samples was negatively associated with metastasis of the lymph node and the suppression of cancer cell invasion and migration. Therefore, we postulated that EFEMP1 may be involved in EMT. In this study, we found that EFEMP1 upregulated the expression of epithelial marker and downregulated the expression of interstitial markers in EC cells and mouse disseminated tumor sections. Notably, EFEMP1 prevented EMT and maintained epithelial morphology during TGF-β-induced EMT. It has been reported that in ovarian cancer and EC, E2 could potentiate tumor progression and invasion through effects on EMT-related markers, including E-cadherin and Snail [30, 31, 32]. Our previous data showed that EFEMP1 suppressed MMP2 and MMP9 secretion in EC and recent studies reported implication of EFEMP1 in the expression of extracellular matrix proteins. For example, overexpression of EFEMP1 reduced the expression of MMP2 and MMP7 [9]. EFEMP1 was a binding pattern of TIPM-3 [33]. Therefore, EFEMP1 may suppress EMT and collaborated with inhibiting secretion of MMPs to promote EC progression and metastasis in EC.

Accumulating evidence suggests that aberrant activation of Wnt/β-catenin signaling is involved in EC development and progression [34, 35]. However, little is known about the association among EFEMP1, EMT and Wnt/β-catenin signaling. The results of this study indicated that EFEMP1-downregulation facilitated EMT and promoted Wnt/ β-catenin signaling then inhibiting the expression of Cyclin-D1 and c-Myc. Furthermore, we also observed that treatment with XAV939 or LiCl reversed the changes in EMT markers induced by EFEMP1 silencing or overexpression, respectively. These results indicate that EFEMP1 suppresses EMT most likely via Wnt/β-catenin signaling in EC cells. In contrast, in light of this involvement of Wnt/β-catenin signaling, Yongyi *et al* [36] reported that E2 activated Wnt/β-catenin signaling and enhanced proliferation during the normal menstrual cycles, whereas progesterone inhibited Wnt/β-catenin signaling to counterbalance E2-induced proliferation and enhance differentiation during the secretory phase. Thus, the unopposed action of E2 may lead to hyperplasia or early endometrial carcinogenesis via Wnt/β-catenin signaling. Therefore, there is a possible cross-talk among E2, EFEMP1, Wnt/ β-catenin pathway and EMT in EC tumorigenesis and development.

In clinic, the most common basis to estimate the prognosis of endometrial carcinoma is the categorization of type I and type II EC. However, the practical value of this approach is limited because up to 20% of type I cancers recur [35, 37]. Although a recent study showed that ERα-negative ECs were associated with increased EMT, which may represent a potential marker for the treatment response to EMT inhibitors [38], there was considerable morphologic and molecular overlap between these two cancer types. Furthermore, in contrast to breast cancer treatment, ERα status has not been used systematically to tailor hormonal therapy or other targeted treatments for EC [39, 40]. So, it is necessary to improve upon the identification of patients at risk for recurrence and poor outcome, and molecular markers may assist in this goal. In both our current and previous study, we observed that the expression of EFEMP1 was decreased in EC due to promoter hypermethylation and E2, which subsequently promoted EMT. Recently, in pleural mesothelioma, it was noted that EFEMP1 could serve as a sensitive and specific biomarker to mesothelioma [41]. Then, the chemical therapy targeting E2 and EFEMP1 may be cooperated to enhance the effect of prognosis of EC patients, although further researches are required to confirm this hypothesis.

In conclusion, the data reported herein reveal that estrogen/ERα signal downregulated the expression of EFEMP1 by directly binding to the promoter of EFEMP1, and EFEMP1 suppresses EMT and migration in EC by inhibiting Wnt/β-catenin signaling. With respect to its tumor suppressor function in EC, EFEMP1 might be an excellent candidate for a therapeutic target in EC.

## MATERIALS AND METHODS

### Samples and patients

The Human Investigation Ethical Committee of the International Peace Maternity and Child Hospital affiliated to Shanghai Jiao Tong University approved this study. Samples of EC and normal endometrial tissues were collected after written informed consent. Primary tumor tissue samples were acquired from 120 endometrial carcinoma patients who underwent hysterectomy with lymph node dissection at our institution between April 2009 and July 2012. Among them, 58 fresh samples were immediately frozen at -80°C for future research. In addition, 50 normal endometrium samples were obtained from patients who underwent hysterectomy due to other diseases than endometrial carcinoma. The stages and histological grades of these tumors were established based on the FIGO criteria (2009; http://www.figo.org/). An independent pathologist verified the histological diagnosis of all collected tissues.

### Cell culture and transfection

The human EC cell lines HEC-1B, RL95-2 and Ishikawa were obtained from the Chinese Academy of Sciences Committee Type Culture Collection (Shanghai, China). Cells were maintained in Dulbecco's modified Eagle's medium (DMEM)/F12 (11030; Gibco, Auckland, NZ) supplemented with 10% fetal bovine serum (FBS; 16000-44; Gibco, Carlsbad, CA) in a humidified atmosphere containing 5% CO_2_ at 37°C. Prior to treatment with 1 μM or 100 nM β-estradiol (E2, Sigma; St. Louis, MO), 1 μM ICI 182,780 (ERα specific antagonist, Tocris; Ellisville, MO) or 1 μM propyl pyrazole triol (PPT, ERα specific agonist, Sigma; St. Louis, MO), cells were cultured in serum-free medium for 24 h to minimize the influence of FBS. To induce EMT, TGF-β (PEPROTECH; NJ, USA) was administered at a concentration of 2ng/ml for 3 days. The Wnt/β-catenin signaling activator LiCl (Sigma; St. Louis, MO) 20mM and inhibitor XAV93920 (Selleck Chemicals, Houston, TX) 10μM treated for 24h. The ERα-expressing plasmid (RG213277) and its control vector (PS10010), the plasmid encoding ERα silencing (sh)RNA (shERa, GI378604) and its control (shNC, TR3008) were all purchased from OriGene Technologies (BeiJing, PRC). Cells were transiently transfected using Lipofectamine 2000 (Invitrogen Life Technologies; USA) according to the manufacturer's protocol. The establishment of HEC-1B-exEFEMP1 and RL95-2-shEFEMP1 were performed as previous description [11].

### Quantitative RT-PCR analysis

Total RNA was extracted using Trizol reagent (Invitrogen Life Technologies; USA). The isolated RNA was reverse transcribed into complementary DNA using the one-step PrimeScript RT reagent Kit (TaKaRa; Dalian, PRC). cDNA was then quantified by real-time PCR using SYBR Premix Ex Taq in an Eppendorf realplexy mastercycler. The primers used for this PCR are shown in Supplementary Table S1. Data analysis employed the 2(−ΔCt) method, and data were obtained in triplicate from three independent experiments.

### Plasmid construction and luciferase reporter assays

A 675-bp fragment of the upstream region of EFEMP1 was amplified from DNA obtained from normal endometrium using the forward primer 5′-CGGGTACCCCATTCTTGCCAACACTTGT-3′ and the reverse primer 5′-TACTCGAGTGGGTCTGATCTGGCGAAGT-3′. The product was cloned directly into the luciferase reporter assay plasmid pGL3-basic (Promega; Madison, WI, USA). Plasmids expressing three mutations in the ERE of the EFEMP1 promoter region were used to determine the role of this regulatory element in EFEMP1 expression (Figure 2A). All plasmids were confirmed by sequencing.

Ishikawa and RL95-2 cells were seeded into 24-well plates on the day prior to transfection. The following day, 200 ng of EFEMP1 promoter reporter-containing plasmid (WT and MUT) together with 200 ng of internal control plasmid expressing Renilla-luciferase were co-transfected using Lipofectamine 2000 (Invitrogen Life Technologies; USA). Reporter activity was measured at 48 h post-transfection using a Dual-Luciferase Assay System (Promega; Madison, WI, USA). Besides, cells were also co-transfected with ERα-expressing plasmid to examine the role of ERα in EFEMP1 expression.

### Chromatin immunoprecipitation (ChIP) assays

ChIP assays were performed according to the manufacturer's protocol (Millipore, USA). Briefly, the assay was performed as followed. Step I: DNA shearing. Cells were fixed in 1% formaldehyde for 10 min and then glycine treated for 5 min before cell scraping and centrifugation. Samples were sonicated to shear DNA to an average fragment size of 300-1500bp. Step II: ChIP.1.7 ml of ChIP Dilution Buffer containing protease inhibitors was added to 300 μl sample and ten percent of each sample was kept as input for PCR. The supernatant was incubated overnight with 5 μg primary antibody ERα and negative control (ChIP grade, Millipore, USA) at 4°C. Subsequently, 60μl of Salmon Sperm DNA/Protein A Agarose was mixed to incubation for 1 hour at 4°C and then carefully removed the supernatant containing unbound, non-specific DNA. The pellet was washed three times on a rotator with washing buffers. Step III: elution, cross-link reversal and DNA purification. The pelleted complex were added 250 μl freshly prepare elution buffer to incubate for 15 min with rotation and collected the supernatant. 20 μl of 5 M NaCl was used to reverse protein-DNA crosslinks of eluates and the input by heating at 65°C for 4 hours. Immunoprecipitates were eluted into 25 μl of TE buffer, and 2 μl of the DNA was used in 20μl PCR reactions using the SYBR Green Mix (TaKaRa; Dalian, PRC). Specific primers used for the ChIP-PCR are shown in Supplementary Table S1. Enrichment was calculated as a percentage of the input. At least two individual repeats of the entire experiment were conducted and data combined to give an average % Input.

### Immunofluorescence

Cells were cultured on glass cover slips and fixed in 4% paraformaldehyde for 30 min. The cells were then permeabilized using 0.25% Triton X-100 for 10 min at room temperature and blocked with 5% bovine serum albumin for 30 min at 37°C. The primary antibodies anti-EFEMP1 (1:200; Abcam, HK), anti-E-cadherin (1:100; CST, USA), anti-Vimentin (1:100; CST), anti-Snail (1:100; CST) and anti-β-catenin (1:100; Epitomics, Burlingame, CA, USA) were incubated with the samples overnight at 4°C. A species-specific secondary rhodamine-labeled antibody (1:200, Epitomics, USA) was incubated with the samples for 30 min at 37°C, and the cells were then counterstained with DAPI at room temperature for 10 min. Control samples were incubated with PBS in place of the primary antibody. Samples were mounted with antifade solution (Tocris; Ellisville, MO) prior to imaging using a laser scanning confocal microscope (Leica TCS-SP5 II, Heidelberg, Germany).

### Immunohistochemistry (IHC) and western blotting

IHC and Western blotting were performed as described previously [11]. The primary antibodies included those used for immunofluorescence studies as well as anti-Cyclin-D1 and anti-c-Myc (both from Epitomics, Burlingame, CA, USA). β-actin was used as a loading control.

### Cell invasion and migration assays

Cell invasion activity was accomplished using BD BioCoat Matrigel Invasion Chambers (BD Biosciences, USA) according to the manufacturer's instructions. Cells (1×10^5^ cells/well) were resuspended in serum-free medium in the upper chamber, which was coated with 40 ml of Matrigel at a 1:3 dilution (BD Biosciences). Medium containing 10% FBS was added to the lower chamber. After being incubated at 37°C for 48 h, cells on the upper side of the membrane were removed using cotton swabs, and cells that were attached to the underside of the membranes were fixed in 4% paraformaldehyde and stained with crystal violet. Five randomly selected fields of view (x100 magnification) were counted to obtain a value for cell invasion. Cell migration assays were performed using transwell chambers not coated with Matrigel, following a similar protocol. Wound healing assays were also conducted to evaluate cellular migration and representative images were captured at the time of wounding and at 24 h after wounding. All experiments were repeated at least three times.

### EC orthotopic tumor and peritoneum metastasis assay in nude mice

4-to 6-week-old female BALB/c mice were purchased from the Shanghai Life Science Institute (Slac Laboratory Animal Co. Ltd, PRC). The animals were raised in pathogen-free conditions. All mice were handled based on the Guidelines for the Care and Use of Laboratory Animals. Surgeries were performed as described previously [20]. Cells (1×10^7^ Ishikawa) were injected subcutaneously into the flanks of nude mice. After 2 weeks, the tumors were removed for orthotopic implantation. A tumor sample of 1 mm^3^ in size was immediately implanted into the posterior face of the uterus and fixed. Half mice (5 mice) were supplemented with estrogen (17β-estradiol 90-day release pellets, 0.72mg/pellet; Innovative Research of America, Toledo, OH). After 4 weeks, these mice were euthanized, and the peritoneal cavity was carefully examined. The tumors were removed, measured and weighed prior to histological evaluation.

Besides, to demonstrate the role of EFEMP1 on tumor metastasis, EFEMP1 stable overexpressing cells (HEC-1B-exEFEMP1) was used to establish the model of peritoneum metastasis. GFP reporter imaging was performed to monitor the cell seeding. After intraperitoneal injection (1×10^7^cells) for 5-6 weeks, the obvious tumors were observed using the NightOWL LB981 NC100 system (Berthold Technologies; Bad Wildbad, Germany). Laparotomy was performed to resect and count disseminated tumors. The volume of the disseminated tumor was measured by the sum of all tumors, and calculated as (Rmax)×(R^2^min)/2.

### Statistical analyses

Statistical analyses were performed using the Statistical Package for the Social Sciences (SPSS) software version 17.0 (Chicago, IL, USA). Data were presented as the mean ± one standard deviation (SD). Continuous variables were examined using unpaired Student's t-tests or one-way ANOVAs (the latter in the case of multiple comparisons). The χ^2^ test was used to examine 2×2 tables, and Spearman tests were used for correlation analyses. Differences were considered statistically significant with an alpha value set to P<0.05.

## SUPPLEMENTARY FIGURE AND TABLES



## References

[1] Siegel R, Naishadham D, Jemal A (2015). Cancer statistics, 2015. CA Cancer J Clin.

[2] Hecht JL, Mutter GL (2006). Molecular and pathologic aspects of endometrial carcinogenesis. J Clin Oncol.

[3] Berstein LM, Tchernobrovkina AE, Gamajunova VB, Kovalevskij AJ, Vasilyev DA, Chepik OF, Turkevitch EA, Tsyrlina EV, Maximov SJ, Ashrafian LA, Thijssen JH (2003). Tumor estrogen content and clinico-morphological and endocrine features of endometrial cancer. J Cancer Res Clin Oncol.

[4] Wong YF, Cheung TH, Lo KW, Yim SF, Siu NS, Chan SC, Ho TW, Wong KW, Yu MY, Wang VW, Li C, Gardner GJ, Bonome T (2007). Identification of molecular markers and signaling pathway in endometrial cancer in Hong Kong Chinese women by genome-wide gene expression profiling. Oncogene.

[5] Timpl R, Sasaki T, Kostka G, Chu ML (2003). Fibulins: a versatile family of extracellular matrix proteins. Nat Rev Mol Cell Biol.

[6] Stone EM, Lotery AJ, Munier FL, Heon E, Piguet B, Guymer RH, Vandenburgh K, Cousin P, Nishimura D, Swiderski RE, Silvestri G, Mackey DA, Hageman GS (1999). A single EFEMP1 mutation associated with both Malattia Leventinese and Doyne honeycomb retinal dystrophy. Nat Genet.

[7] Seeliger H, Camaj P, Ischenko I, Kleespies A, De Toni EN, Thieme SE, Blum H, Assmann G, Jauch KW, Bruns CJ (2009). EFEMP1 expression promotes in vivo tumor growth in human pancreatic adenocarcinoma. Mol Cancer Res.

[8] Song EL, Hou YP, Yu SP, Chen SG, Huang JT, Luo T, Kong LP, Xu J, Wang HQ (2011). EFEMP1 expression promotes angiogenesis and accelerates the growth of cervical cancer in vivo. Gynecol Oncol.

[9] Kim EJ, Lee SY, Woo MK, Choi SI, Kim TR, Kim MJ, Kim KC, Cho EW, Kim IG (2012). Fibulin-3 promoter methylation alters the invasive behavior of non-small cell lung cancer cell lines via MMP-7 and MMP-2 regulation. Int J Oncol.

[10] Sadr-Nabavi A, Ramser J, Volkmann J, Naehrig J, Wiesmann F, Betz B, Hellebrand H, Engert S, Seitz S, Kreutzfeld R, Sasaki T, Arnold N, Schmutzler R (2009). Decreased expression of angiogenesis antagonist EFEMP1 in sporadic breast cancer is caused by aberrant promoter methylation and points to an impact of EFEMP1 as molecular biomarker. Int J Cancer.

[11] Yang T, Qiu H, Bao W, Li B, Lu C, Du G, Luo X, Wang L, Wan X (2013). Epigenetic inactivation of EFEMP1 is associated with tumor suppressive function in endometrial carcinoma. PLoS One.

[12] Blackburn J, Tarttelin EE, Gregory-Evans CY, Moosajee M, Gregory-Evans K (2003). Transcriptional regulation and expression of the dominant drusen gene FBLN3 (EFEMP1) in mammalian retina. Invest Ophthalmol Vis Sci.

[13] Lee JM, Dedhar S, Kalluri R, Thompson EW (2006). The epithelial-mesenchymal transition: new insights in signaling, development, and disease. J Cell Biol.

[14] Thiery JP, Acloque H, Huang RY, Nieto MA (2009). Epithelial-mesenchymal transitions in development and disease. Cell.

[15] Kalluri R, Weinberg RA (2009). The basics of epithelial-mesenchymal transition. J Clin Invest.

[16] Yang J, Weinberg RA (2008). Epithelial-mesenchymal transition: at the crossroads of development and tumor metastasis. Dev Cell.

[17] Valenta T, Hausmann G, Basler K (2012). The many faces and functions of beta-catenin. EMBO J.

[18] Huang J, Xiao D, Li G, Ma J, Chen P, Yuan W, Hou F, Ge J, Zhong M, Tang Y, Xia X, Chen Z (2013). EphA2 promotes epithelial-mesenchymal transition through the Wnt/beta-catenin pathway in gastric cancer cells. Oncogene.

[19] Schiemann WP, Blobe GC, Kalume DE, Pandey A, Lodish HF (2002). Context-specific effects of fibulin-5 (DANCE/EVEC) on cell proliferation, motility, and invasion. Fibulin-5 is induced by transforming growth factor-beta and affects protein kinase cascades. J Biol Chem.

[20] Che Q, Liu BY, Liao Y, Zhang HJ, Yang TT, He YY, Xia YH, Lu W, He XY, Chen Z, Wang FY, Wan XP (2013). Activation of a positive feedback loop involving IL-6 and aromatase promotes intratumoral 17beta-estradiol biosynthesis in endometrial carcinoma microenvironment. Int J Cancer.

[21] Santen RJ, Lobenhofer EK, Afshari CA, Bao Y, Song RX (2005). Adaptation of estrogen-regulated genes in long-term estradiol deprived MCF-7 breast cancer cells. Breast Cancer Res Treat.

[22] Malik S, Jiang S, Garee JP, Verdin E, Lee AV, O'Malley BW, Zhang M, Belaguli NS, Oesterreich S (2010). Histone deacetylase 7 and FoxA1 in estrogen-mediated repression of RPRM. Mol Cell Biol.

[23] Stossi F, Likhite VS, Katzenellenbogen JA, Katzenellenbogen BS (2006). Estrogen-occupied estrogen receptor represses cyclin G2 gene expression and recruits a repressor complex at the cyclin G2 promoter. J Biol Chem.

[24] Hall J.M, Couse J.F, Korach K.S (2001). The multifaceted mechanisms of estradiol and estrogen receptor signaling. J Biol Chem.

[25] Carroll JS, Meyer CA, Song J, Li W, Geistlinger TR, Eeckhoute J, Brodsky AS, Keeton EK, Fertuck KC, Hall GF, Wang Q, Bekiranov S, Sementchenko V (2006). Genome-wide analysis of estrogen receptor binding sites. Nat Genet.

[26] Kalaitzidis D, Gilmore TD (2005). Transcription factor cross-talk: the estrogen receptor and NF-kappaB. Trends Endocrinol Metab.

[27] Hurtado A, Holmes KA, Geistlinger TR, Hutcheson IR, Nicholson RI, Brown M, Jiang J, Howat WJ, Ali S, Carroll JS (2008). Regulation of ERBB2 by oestrogen receptor-PAX2 determines response to tamoxifen. Nature.

[28] Zhu P, Baek SH, Bourk EM, Ohgi KA, Garcia-Bassets I, Sanjo H, Akira S, Kotol PF, Glass CK, Rosenfeld MG, Rose DW (2006). Macrophage/cancer cell interactions mediate hormone resistance by a nuclear receptor derepression pathway. Cell.

[29] Thiery JP, Sleeman JP (2006). Complex networks orchestrate epithelial-mesenchymal transitions. Nat Rev Mol Cell Biol.

[30] Park SH, Cheung LW, Wong AS, Leung PC (2008). Estrogen regulates Snail and Slug in the down-regulation of E-cadherin and induces metastatic potential of ovarian cancer cells through estrogen receptor alpha. Mol Endocrinol.

[31] Gentilini D, Busacca M, Di Francesco S, Vignali M, Vigano P, Di Blasio AM (2007). PI3K/Akt and ERK1/2 signalling pathways are involved in endometrial cell migration induced by 17beta-estradiol and growth factors. Mol Hum Reprod.

[32] Acconcia F, Barnes CJ, Kumar R (2006). Estrogen and tamoxifen induce cytoskeletal remodeling and migration in endometrial cancer cells. Endocrinology.

[33] Klenotic PA, Munier FL, Marmorstein LY, Anand-Apte B (2004). Tissue inhibitor of metalloproteinases-3 (TIMP-3) is a binding partner of epithelial growth factor-containing fibulin-like extracellular matrix protein 1 (EFEMP1). Implications for macular degenerations. J Biol Chem.

[34] Matias-Guiu X, Prat J (2013). Molecular pathology of endometrial carcinoma. Histopathology.

[35] Yeramian A, Moreno-Bueno G, Dolcet X, Catasus L, Abal M, Colas E, Reventos J, Palacios J, Prat J, Matias-Guiu X (2012). Endometrial carcinoma: molecular alterations involved in tumor development and progression. Oncogene.

[36] Wang Y, Hanifi-Moghaddam P, Hanekamp EE, Kloosterboer HJ, Franken P, Veldscholte J, van Doorn HC, Ewing PC, Kim JJ, Grootegoed JA, Burger CW, Fodde R, Blok LJ (2009). Progesterone inhibition of Wnt/beta-catenin signaling in normal endometrium and endometrial cancer. Clin Cancer Res.

[37] Bokhman JV (1983). Two pathogenetic types of endometrial carcinoma. Gynecol Oncol.

[38] Wik E, Raeder MB, Krakstad C, Trovik J, Birkeland E, Hoivik EA, Mjos S, Werner HM, Mannelqvist M, Stefansson IM, Oyan AM, Kalland KH, Akslen LA (2013). Lack of estrogen receptor-alpha is associated with epithelial-mesenchymal transition and PI3K alterations in endometrial carcinoma. Clin Cancer Res.

[39] Decruze SB, Green JA (2007). Hormone therapy in advanced and recurrent endometrial cancer: a systematic review. Int J Gynecol Cancer.

[40] Kokka F, Brockbank E, Oram D, Gallagher C, Bryant A (2010). Hormonal therapy in advanced or recurrent endometrial cancer. Cochrane Database Syst Rev.

[41] Pass HI, Levin SM, Harbut MR, Melamed J, Chiriboga L, Donington J, Huflejt M, Carbone M, Chia D, Goodglick L, Goodman GE, Thornquist MD, Liu G (2012). Fibulin-3 as a blood and effusion biomarker for pleural mesothelioma. N Engl J Med.

