# Supplementary material for: EFEMP1 is repressed by estrogen and inhibits the epithelial-mesenchymal transition via Wnt/β-catenin signaling in endometrial carcinoma
Source: Oncotarget. 2016 Mar 22;7(18):25712–25. doi: 10.18632/oncotarget.8263 (PMC5041938; doi:10.18632/oncotarget.8263)
Supplement: Supplementary file 1 [file oncotarget-07-25712-s001.pdf]

SUPPLEMENTARY FIGURE AND TABLES

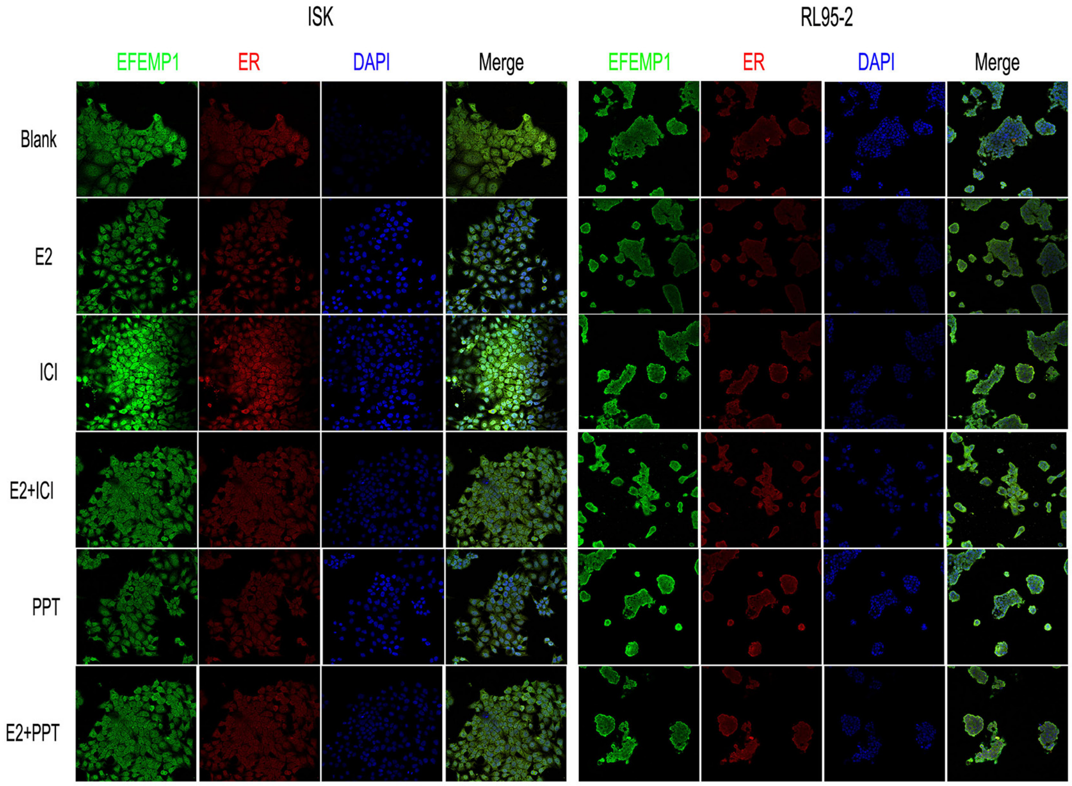

Figure S1: Immunofluorescence analysis of the effect of E2, ICI and/or PPT on the expression of EFEMP1. Ishikawa and RL95-2 cells were treated with E2 (10^{-6} M and 10^{-7} M, respectively), ICI (10^{-6} M) and/or PPT (10^{-6} M) for 24 h. *Red* denotes EFEMP1; *Green* denotes ER á; *Blue* denotes DAPI-stained nuclei. All images were obtained on the same day using the same microscope settings. Original magnification, ×400.

Supplementary Table 1: The clinic-pathological variables of 120 endometrial carcinoma tissues

Variable	No. patients (%)	
	n	%
Total	120	100
Age (years)		
≤50	65	54.2
>50	55	45.8
FIGO stage		
Stage I	69	57.5
Stage II	36	30
Stage III	15	12.5
Grade		
G1	58	48.3
G2	42	35
G3	20	16.7
Histological type		
Endometrioid	96	80
Nonendometriod	24	20
Myometrial invasion		
≤1/2	85	70.8
>1/2	35	29.2
Lymph node metastasis		
No (N-)	83	69.2
Yes(N+)	37	30.8
Lymphovascula space involvement		
No	76	63.3
Yes	44	36.7
ER expression		
Negative	34	28.3
Positive	86	71.7
PR expression		
Negative	28	23.3
Positive	92	76.7

Supplementary Table 2: Primers used for real-time PCR analysis

Gene	Primer sequence
EFEMP1	Forward: 5’-CAGGACACCGAAGAAACCAT-3’
	Reverse : 5’-GTTTCCTGCTGAGGCTGTTC-3’
ESR1(ERα)	Forward: 5’-TGATTGGTCTCGTCTGGCG-3’
	Reverse : 5’-CATGCCCTCTACACATTTTCCC-3’
E-cadherin	Forward: 5’-TTGCTACTGGAACAGGGACAC-3’
	Reverse : 5’-CCCGTGTGTTAGTTCTGCTGT-3’
Vimentin	Forward: 5’-TGCGTGAAATGGAAGAGAACT-3’
	Reverse : 5’-TCAGGTTCAGGGAGGAAAAGT-3’
Snail	Forward: 5’-TCCAGAGTTTACCTTCCAGCA-3’
	Reverse : 5’-CTTTCCCACTGTCCTCATCTG-3’
β-catenin	Forward: 5’-TGCTGAAGGTGCTATCTGTCTG-3’
	Reverse : 5’-TCCATCCCTTCCTGTTTAGTTG-3’
Cyclin-D1	Forward: 5’-GCCCTCGGTGTCCTACTTCA-3’
	Reverse : 5’-AAGACCTCCTCCTCGCACTTCT-3’
c-Myc	Forward: 5’-CCTCCACTCGGAAGGACTATC-3’
	Reverse : 5’-GTGTTCGCCTCTTGACATTCTC-3’
β-actin	Forward: 5’-CAGCCATGTACGTTGCTATCCAGG-3’
	Reverse : 5’-AGGTCCAGACGCAGGATGGCATG-3’
Primers for CHIP	Forward: 5’-TGAAGCGCATCCAGGACATC-3’
	Reverse : 5’-TGAGCCCAGCGTTGCGAGCC-3’
